# Substantively Lowered Levels of Pantothenic Acid (Vitamin B5) in Several Regions of the Human Brain in Parkinson’s Disease Dementia

**DOI:** 10.3390/metabo11090569

**Published:** 2021-08-25

**Authors:** Melissa Scholefield, Stephanie J. Church, Jingshu Xu, Stefano Patassini, Nigel M. Hooper, Richard D. Unwin, Garth J. S. Cooper

**Affiliations:** 1 Centre for Advanced Discovery & Experimental Therapeutics, Division of Cardiovascular Sciences, School of Medical Sciences, Faculty of Biology, Medicine and Health, The University of Manchester, Manchester M13 9NT, UK; stephanie.church@manchester.ac.uk (S.J.C.); jingshu.xu@hotmail.com (J.X.); R.Unwin@manchester.ac.uk (R.D.U.); 2 School of Biological Sciences, Faculty of Science, University of Auckland, Private Bag 92 019, Auckland 1142, New Zealand; stefano_patas@yahoo.it; 3 Division of Neuroscience & Experimental Psychology, School of Biological Sciences, Faculty of Biology, Medicine and Health, The University of Manchester, Manchester M13 9NT, UK; nigel.hooper@manchester.ac.uk; 4 Stoller Biomarker Discovery Centre & Division of Cancer Sciences, School of Medical Sciences, Faculty of Biology, Medicine and Health, The University of Manchester, Citylabs 1.0 (Third Floor), Nelson Street, Manchester M13 9NQ, UK

**Keywords:** pantothenic acid, vitamin B5, Parkinson’s disease dementia, mass spectrometry, Alzheimer’s disease, Huntington’s disease, metabolomics

## Abstract

Pantothenic acid (vitamin B5) is an essential trace nutrient required for the synthesis of coenzyme A (CoA). It has previously been shown that pantothenic acid is significantly decreased in multiple brain regions in both Alzheimer’s disease (ADD) and Huntington’s disease (HD). The current investigation aimed to determine whether similar changes are also present in cases of Parkinson’s disease dementia (PDD), another age-related neurodegenerative condition, and whether such perturbations might occur in similar regions in these apparently different diseases. Brain tissue was obtained from nine confirmed cases of PDD and nine controls with a post-mortem delay of 26 h or less. Tissues were acquired from nine regions that show high, moderate, or low levels of neurodegeneration in PDD: the cerebellum, motor cortex, primary visual cortex, hippocampus, substantia nigra, middle temporal gyrus, medulla oblongata, cingulate gyrus, and pons. A targeted ultra–high performance liquid chromatography—tandem mass spectrometry (UHPLC-MS/MS) approach was used to quantify pantothenic acid in these tissues. Pantothenic acid was significantly decreased in the cerebellum (*p* = 0.008), substantia nigra (*p* = 0.02), and medulla (*p* = 0.008) of PDD cases. These findings mirror the significant decreases in the cerebellum of both ADD and HD cases, as well as the substantia nigra, putamen, middle frontal gyrus, and entorhinal cortex of HD cases, and motor cortex, primary visual cortex, hippocampus, middle temporal gyrus, cingulate gyrus, and entorhinal cortex of ADD cases. Taken together, these observations indicate a common but regionally selective disruption of pantothenic acid levels across PDD, ADD, and HD.

## 1. Introduction

Parkinson’s disease (PD) is a common neurodegenerative disorder, primarily characterised clinically by bradykinesia, resting tremor, and rigidity, and neuropathologically by extensive dopaminergic neuronal loss and accumulation of α-synuclein deposits known as Lewy bodies. These changes occur most severely within the substantia nigra pars compacta, but also throughout other regions of the brain [1,2,3]. As well as motor dysfunction, cognitive impairment is a common symptom in PD as the disease progresses, with up to 46% of individuals going on to develop Parkinson’s disease dementia (PDD) in the ten years following initial diagnosis [4], and up to 80% by twenty years [5].

Despite how common the condition is, the causes and mechanisms of PDD remain poorly characterised. Most cases are sporadic with no identifiable cause, and treatments remain purely symptomatic, with even the most promising clinical trials thus far being unable to slow, stop, or reverse the progression of the disease. This is also the case with other similar neurodegenerative diseases, such as Alzheimer’s (ADD) and Huntington’s disease (HD), which also currently have only symptomatic treatments despite extensive interest and studies into causes, mechanisms, and potential treatments [6].

These neurodegenerative diseases share several characteristics. Age is a primary risk factor for the development of symptoms (although HD is always caused by a dominant autosomal mutation in the huntingtin gene; the development of symptomology generally occurs with older age), cerebral protein deposition is present in each condition (α-synuclein in PD, tau and amyloid-β in ADD, and huntingtin in HD), and the clinical presentation can show significant overlap, with increased risk of psychological complaints [7], issues with sleep [8], and difficulty walking [9], as well as the progressive development of cognitive impairment [10,11].

These findings have raised the question as to whether there may be common pathogenic insults present across multiple neurodegenerative diseases contributing to these similarities in presentation. Common disruptions in multiple metabolic pathways have already been identified in ADD and HD brains, including widespread urea [12,13,14,15] and glucose increases [13,16,17,18], dysregulation of glucose and purine metabolism pathways [13,16,17,19,20], and decreases in the essential nutrient pantothenic acid [21,22], also known as vitamin B5. Pantothenic acid is essential for the synthesis of coenzyme A (CoA), a molecule with extensive roles in metabolism including in the tricarboxylic acid (TCA) cycle, fatty acid metabolism, and acetylcholine and myelin synthesis, amongst others. Dysregulation of pantothenic acid could disrupt the supply of this essential molecule, with widespread downstream metabolic complications. Inborn mutations in the pantothenate kinase 2 gene (PKAN2), which is an intermediate in the CoA synthesis pathway, are associated with a disease known as pantothenate kinase-associated neurodegeneration [23], a condition characterised by severe brain damage within the basal ganglia and progressive cognitive and motor dysfunction including parkinsonism.

Levels of pantothenic acid have not, to our knowledge, been investigated previously in the PDD brain. Thus, this investigation is, to our knowledge, the first to report dysregulation of cerebral pantothenic acid in PDD, similar to that previously observed in ADD and HD.

## 2. Results

### 2.1. Cohort Characterisation

Cases and controls were matched for age, sex, post-mortem delay (PMD), and brain weight, with no significant differences in any of these variables (see Table 1). Samples were acquired with the lowest PMD possible, with a maximum of 26 h. Additional metadata, including cause of death and disease staging, were also obtained for all samples (see Supplementary Material A).

Due to a lack of available SN tissue for two controls, two additional, replacement SN samples (C10 and C11) were obtained from different donors to those employed for the other brain regions. When the revised SN cohort was analysed, it was still matched for age, sex, and brain weight, but cases had a lower PMD than controls (*p* = 0.03; see Table 1).

### 2.2. Pantothenic Acid Analysis

**Figure 1:**
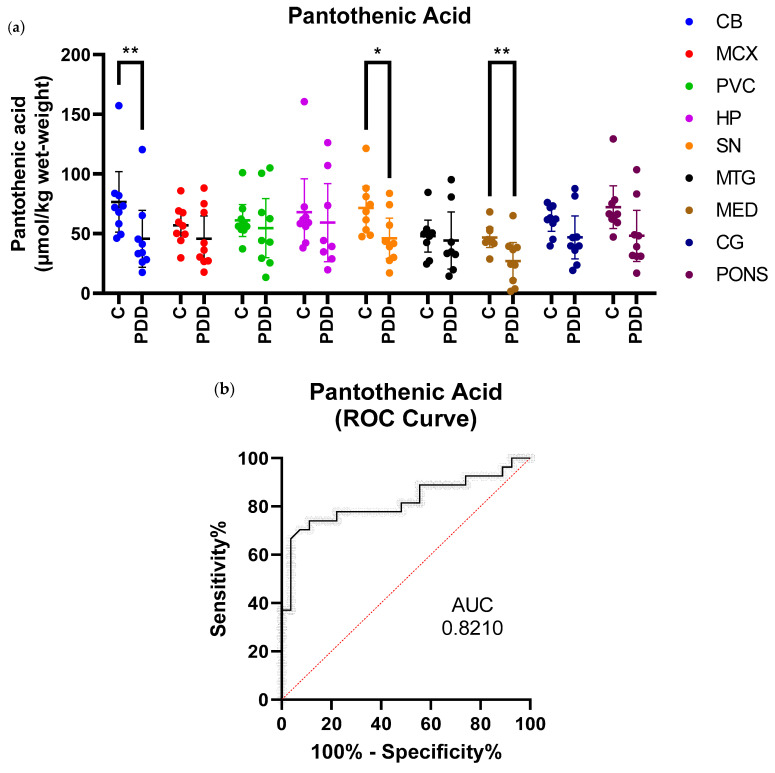
Pantothenic Acid Concentrations in PDD Cases and Controls. (**a**) Mean pantothenic acid concentrations ± 95% confidence intervals in µmol/kg wet-weight. Case–control differences were determined using Mann–Whitney *U* test. * *p* < 0.05; ** *p* < 0.01. C = Controls; PDD = PDD cases. (**b**) receiver-operating characteristic (ROC) curve for pantothenic acid in all brain regions showing significant case–control differences (SN, CB, and MED). Area under curve (AUC) value was 0.8210 with *p* < 0.0001.

Concentrations of pantothenic acid were found to be significantly lower in the CB (*p* = 0.008), SN (*p* = 0.02), and MED (*p* = 0.008) of cases compared to controls (see Table 2 and Figure 1a). In these three regions, there was a decrease of approximately 40% in PDD cases. There was also a suggestion of decreased pantothenic acid in the pons, but this did not reach significance (*p* = 0.0503). Inter-regional concentrations of pantothenic acid were consistent, with no significant differences between any two regions in either cases or controls (data not shown). A ROC curve was generated using values from all three regions showing significant case–control differences (SN, CB, and MED; see Figure 1b). The ROC curve has an area under curve (AUC) value of 0.82 (*p* < 0.0001), indicating good discriminatory power in distinguishing between PDD cases and controls.

Neither of the substituted SN controls showed significant differences in pantothenic acid concentrations compared to the other cases in the SN cohort (see Supplementary Material B for individual values). Case–control differences in the SN remained significant with exclusion of substituted SN controls C10 and C11. A previous analysis by our group of the effects of PMD on human and rat brain metabolites found no changes in pantothenic acid concentrations up to 24 h PMD [24]. Together with the data here, there appears to be no significant effect of PMD on pantothenic acid levels in the current cohort.

Statistical analyses were performed following this analysis to confirm the sample size used was sufficient to determine significant case–control differences at *p* < 0.05: the required sample size was <10 in all regions that showed significant case–control differences, as well as in the CG and pons (see Supplementary Table S3).

### 2.3. Comparisons with ADD and HD

**Figure 2:**
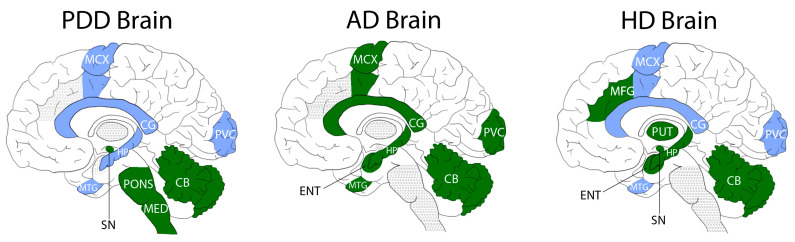
Regional Distribution of Pantothenic Acid Decreases in the PDD, ADD, and HD Brain. Areas shaded in green denote regions with significant decreases in pantothenic acid compared to matched cohort controls. Areas shaded in blue denote regions showing no significant case–control differences. Areas shaded in grey denote regions that were not investigated.

As well as investigating case–control comparisons of pantothenic acid in PDD, we also compared the current results with those we obtained in previous analyses of other neurodegenerative diseases. Our group has previously performed analyses of pantothenic levels in ADD [21] and HD [17,22] by methods comparable to those used in this study. Although there is some variation in the brain regions investigated, it is possible to compare findings across multiple areas of the brain in all three conditions, including the CB, MCX, PVC, HP, MTG, and CG, as well as the SN in PDD and HD (see Table 3 and Figure 2).

Notably, PDD, ADD, and HD all showed significantly decreased pantothenic acid in the CB, where the reduction was ~40–50%. This is the only region investigated in all three diseases that showed a significant alteration in every condition. Both PDD and HD show a significant reduction of approximately 40% in the SN, but this region was not investigated in ADD. The MED was not included in either the ADD or HD studies, and so cannot be compared to PDD. Both ADD and HD showed significantly decreased pantothenic acid in the entorhinal cortex (ENT), but this region was not investigated in the current PDD study.

Pantothenic acid dysregulations appear to be less widespread in PDD than either ADD or HD, showing changes in three of nine investigated regions, in comparison to seven of eleven regions in HD and all seven areas reported on in ADD. Where reductions occur, they are on a similar scale, at approximately 60% of control values.

## 3. Discussion

Outside our own previous analyses, pantothenic acid levels have not, to our knowledge, been investigated in the ADD, HD, or PDD brain. Interestingly though, an increased dietary intake of pantothenic acid has been associated with increased amyloid-β burden in individuals with cognitive impairment [25]. This may indicate that pantothenic acid deficiencies in the brain cannot be counteracted by an increased dietary intake. In PD, increased microbial pantothenic acid production in patient stool samples has been positively associated with non-motor symptoms such as constipation by one group [26], whereas another investigation has reported decreased pantothenic acid levels in PD patient faecal samples [27]. Altogether, the current literature on pantothenic acid levels in dementia is, at present, very limited, and the current investigation offers novel insights into disruptions in this molecule within the PDD brain.

The cohort for this investigation was carefully selected to avoid as many possible confounding factors as possible, including close matching for age, sex, PMD, and brain weight. A PMD of 26 h or less was considered suitable, as a previous analysis carried out by our group has observed no changes in pantothenic acid concentrations in either the cerebellum or cortex of rats for up to 24 h PMD, although a modest–significant increase was observed by 48 h in the cortex [24]. To our knowledge, this has not been investigated in human brains, most likely due to the high number of confounding factors that would make getting sufficient sample sizes difficult, and the unfeasibility of obtaining very low PMD brain samples from humans. As such, we take the results from the investigation on rat brains to be the best available evidence for our selected PMD timeframe but are cognisant that human brain samples may show differences in this respect to animal models.

Pantothenic acid is required for synthesis of CoA, which plays roles in many major metabolic pathways, including several that have previously been reported to be disrupted in PD. For example, CoA is a carbon transporter in the TCA cycle, which has been reported to show downregulated gene expression and enzyme levels in PD brain [28,29], as well as decreased metabolite intermediate levels in the PD cerebrospinal fluid [30]. Reductions in pantothenic acid may lead to insufficient production of CoA for proper functioning of the TCA cycle.

It is possible that such a perturbation may converge with disruptions in other metabolic pathways. Usually, pyruvate produced by the breakdown of glucose in the glycolytic pathway would enter the TCA cycle after being converted into acetyl-CoA in the mitochondria. This process itself requires the presence of CoA. Glycolysis has been widely observed to be downregulated in PD [31], and even suggested as a possible therapeutic target in the disease [32]. This dysregulation reflects increased levels of glucose [33] and overall glucose hypometabolism throughout the PD and PDD brain [31,34,35], correlating with motor and cognitive symptoms [36,37]. As such, reduced pantothenic acid may link not only reduced TCA cycle activity, but also decreased glycolysis and glucose hypometabolism in the PDD brain. This may be a shared pathogenic mechanism with ADD and HD, which also show increased brain-glucose levels, impaired glycolytic and TCA cycle activity, and increased glucose hypometabolism throughout the brain [12,13,16,17,18,20].

In order to determine whether these changes are initial drivers of metabolic dysfunction in PDD, rather than simply being a late-stage downstream result of neuronal cell death, it is necessary to quantify pantothenic acid levels in pre-symptomatic or early-stage PD/D patients. However, as there are currently no methods available for quantifying pantothenic acid in vivo to the authors’ knowledge, and as it is not yet possible to determine who will go on to develop PD with certainty (and so take measurements from pre-symptomatic individuals who passed away prior to developing the disease), this cannot be investigated at this stage. However, pantothenic acid has been seen to be decreased in the brains of individuals with pre-symptomatic HD, which can be positively diagnosed due to the autosomal dominant huntingtin mutation present in all individuals with HD from birth, with levels most markedly lowered in highly impacted brain regions [38]. This indicates that pantothenic acid levels can decrease prior to extensive neuronal loss, but development of in vivo techniques for measuring pantothenic acid or reliable biomarkers for diagnosing pre-symptomatic PD would be necessary to conclude that this is also the case in PD/D.

Another possible contributor to the alterations observed here could be decreased microbial production of pantothenic acid. Over recent decades, more and more studies have been reporting observations that support the microbiota–gut–brain hypothesis of PD, wherein microbial alterations and initial accumulations of pathogenic α-synuclein deposits in the gut spread in a prion-like manner from the peripheral to the central nervous system, resulting in inflammation, increased permeability of the blood–gut and blood–brain barriers, and the spread of α-synuclein inclusions to the PD brain itself [39,40,41]—in some cases, before the development of motor symptoms [42]. Indeed, α-synuclein Braak staging itself, commonly used to determine the PD disease stage, has described α-synuclein inclusions in the gastrointestinal tract of PD patients [43]. Decreased microbial production of pantothenic acid has been observed in stool samples taken from PD patients, and has been associated with non-motor symptoms, particularly constipation [26], which is commonly observed in pre-symptomatic individuals that later go on to develop PD [44]. Another study has also reported overall decreases in pantothenic acid levels in the PD gut microbiota taken from faecal samples along with reductions in anti-inflammatory bacteria [27]. If faecal samples could be obtained from individuals who later go on to develop PD for quantification of pantothenic acid, it could be better determined whether pantothenic acid is likely to be a driving force in metabolic dysregulation in PD/D, or whether it is a later downstream effect of the disease; however, this remains difficult without biomarkers of pre-symptomatic disease.

One interesting distinction between PDD and ADD/HD is the variability in regions affected by lowered pantothenic acid levels. In PDD, only the SN, MED, and CB showed significant reductions in pantothenic acid levels. The SN is the region most severely affected by neuronal loss in PD, and the MED one of the first areas to show Lewy body deposition according to typical PD/PDD progression as defined by α-synuclein Braak staging [1], suggesting that more severely or earlier-affected areas of the PDD brain may show pantothenic acid reduction. However, the CB is not an area typically associated with PD, with varying reports on the degree of atrophy in this region [45], although it has been observed to show functional and morphological changes, which have been theorised to include both pathological and protective functions in the disease [46]. Furthermore, reduced pantothenic acid was also observed in the CB of the ADD and HD cases, indicating a shared perturbation across all three diseases in this region. This may indicate a higher degree of involvement of the cerebellum in neurodegenerative diseases than previously considered and would benefit from further investigation with a larger sample size. HD also showed significant decreases in the SN, but otherwise, there were no regional similarities observed between PDD and ADD or HD. This could suggest a regional susceptibility in pantothenic acid reductions that contributes to the variability in clinical presentations in different conditions, despite some areas of shared pathology and/or symptomology.

However, investigations of other regions of the brain that are highly impacted by neurodegeneration in both PD/D and HD, such as the putamen and caudate nucleus—which were not included in this analysis as regions were selected to try to cover moderately-affected and relatively spared regions of the PDD brain as well as highly affected areas—may reveal more similarities between these conditions than can be observed here. Wider investigation of further highly affected, moderately affected, and relatively spared regions throughout the PDD brain could also provide more evidence towards the possibility of regional susceptibility in this particular disease, which is currently limited here by sample size and the small number of investigated regions. Yet, further to this, the determination of α-synuclein levels within individual brain regions alongside pantothenic acid concentrations could more convincingly determine any correlation between Lewy body deposition and changes in pantothenic acid in the PDD brain. This was not possible with the current sample set but could provide evidence towards the hypothesis of regional susceptibility to pantothenic acid alternations in PDD suggested here.

Overall, pantothenic acid reductions present a novel and region-specific pathogenic insult in PDD, ADD, and HD, which may contribute to observed disease mechanisms such as glucose hypometabolism and other related metabolic dysfunctions.

## 4. Materials and Methods

### 4.1. Tissue for Pantothenic Acid Quantification

Brain tissue from nine regions, including the middle temporal gyrus (MTG); motor cortex (MCX); primary visual cortex (PVC); hippocampus (HP); anterior cingulate gyrus (CG); cerebellum, at the level of the dentate nucleus (CB); substantia nigra (SN); pons; and medulla oblongata (MED), were obtained from nine confirmed cases of PDD and nine controls from the University of Miami Brain Endowment Bank, USA (part of the National Institute of Health NeuroBioBank network). These regions were selected based on three main criteria: (1) availability of as many regions as possible from the same donor (this is more difficult when including multiple high-impact regions, which are in high demand), (2) inclusion of not only regions highly impacted in PDD, but also moderately impacted and relatively spared regions in order to investigate whether changes in B5 mirror typical levels of neurodegeneration observed in the PDD brain, and (3) overlap with regions investigated in previous analyses of AD and HD brains, so that comparisons could be made between different conditions. All available patient data were collected and recorded, including age at death, sex, brain weight, post-mortem delay (PMD), α-synuclein Braak stage, and cause of death (see Supplementary Material A for individual patient data).

### 4.2. Diagnosis and Severity of PDD Cases

Tissues obtained from both cases and controls were examined and diagnosed by the referring neuropathologists of the Miami Brain Endowment Bank. All were diagnosed to be of the α-synucleinopathy neocortical type, consistent with the clinical phenotype of PDD, and controls did not show any features of neurodegeneration or vascular pathology. PDD staging was assessed using either Braak staging [1] or McKeith’s typing of Lewy body disease [47]. Individual donor data can be found in Supplementary Material A.

### 4.3. Pantothenic Acid Quantification

Pantothenic acid was quantified in brain extracts by UHPLC-MS/MS. Samples were extracted into 50:50 (*v*/*v*) methanol:chloroform containing 1 µM labelled pantothenic acid standard ((di-β-alanine-^13^C_6_, ^15^N_2_) calcium salt ≥98 atom %, ≥97% (CP); Sigma-Aldrich, St. Louis, MO, USA). Methanol:chloroform:internal standard blanks were also prepared. Samples were lysed in a TissueLyser batch bead homogeniser (Qiagen, Manchester, UK) using carbamide beads. LC-MS grade water was added to lysed samples before centrifugation at 2400× *g* for 15 min to separate polar and non-polar phases. The polar methanol phase was transferred to a fresh tube before drying overnight in a Speedvac centrifugal concentrator (Savant Speedvac, Thermo Scientific, Waltham, MA, USA).

Following drying, 0.1% formic acid was added to each sample and blank. This solution was then transferred to 300 µL autosampler vials (Thermo Fisher Scientific, Waltham, MA, USA). Four blanks containing only 0.1% (*v*/*v*) formic acid were also prepared and interleaved throughout the UHPLC-MS/MS run. Standard solutions containing the labelled pantothenic acid internal standard and unlabelled pantothenic acid external standards (D-Pantothenic acid hemicalcium salt ≥98.0%; Sigma-Aldrich, St. Louis, MO, USA) in 0.1% formic acid were prepared in 300 µL autosampler vials containing concentrations of 0–5000 mM pantothenic acid. These were used to create calibration curves during analysis (see Supplementary Material A). Three QC samples containing 20, 200, and 2000 mM unlabelled pantothenic acid standards in 0.1% (*v*/*v*) formic acid were also prepared and interleaved throughout the run.

Pantothenic acid quantification was performed on a TSQ Vantage triple quadrupole mass spectrometer coupled with an Accela UHPLC system (Thermo Fisher Scientific, Waltham, MA, USA) using a Hypersil Gold AQ column with a diameter of 2.1 mm, length of 100 mm, and particle size of 1.9 µm in reverse-phase mode (Thermo Fisher Scientific 25302-101130, Waltham, MA, USA) and 0.5 µm pre-column filter (Thermo Fisher Scientific 22016, Waltham, MA, USA). The column was maintained at 25 °C during each run. Gradient elution was performed using 0.1% formic acid in water (A) and 0.1% formic acid in acetonitrile (B) at 300 µL/min. Two regions were analysed per run, with randomisation of cases and controls.

### 4.4. UHPLC-MS/MS Data Analysis

Peaks were identified based on the expected retention time (RT) derived by comparison with the RT of the spiked pantothenic acid-labelled internal standard. Peaks were manually checked to ensure correct identification by the software. Standards were only accepted when showing a % difference of <15% of expected, with 6/10 standards required for acceptance of the standard curve. QC samples showing a % difference >20% of expected concentration were excluded, with 2/3 successful QC runs required in each batch for the run to be accepted.

Concentrations of pantothenic acid in each sample were determined based on the calibration curve for each region (see Supplementary Material A). Concentrations were corrected for sample wet-weight and case–control differences analysed in GraphPad Prism v8.1.2. (Prism; La Jolla, CA, USA). A non-parametric Mann–Whitney U test was used due to the small sample size, and a *p*-value < 0.05 was considered significant. A ROC curve was generated by combining all measurements from regions showing significant case–control differences in pantothenic acid (SN, CB, and MED) using GraphPad Prism v8.1.2.

The minimum sample size required to confidently determine case–control differences at a significance level of *p* < 0.05 was calculated using the sample size calculator from SPH Analytics, Alpharetta, GA, USA (https://www.sphanalytics.com/sample-size-calculator-using-average-values/, accessed on 19 February 2021).

The results obtained were compared to those previously reported in both ADD and HD.

## Figures and Tables

**Table 1:** Comparison of Brain Bank Groups.

	Gender (% Male)	Age at Death (Years)	PMD (Hours)
Controls (n = 9)	44	70 (61–79)	19.8 (12.5–26.0)
SN Controls (n = 9) †	44	70 (62–79)	20.6 (10.8–26.0)
Cases (n = 9) †	66	73 (61–81)	14.6 (4.3–21.9) *

Mean (range) age, PMD, brain weight and Braak stage. * *p* < 0.05 between cases and controls as determined by Mann–Whitney U Test. † Brain weight not available for one sample. See Supplementary Material A for individual data.

**Table 2:** Pantothenic Acid Concentrations in PDD Cases and Controls.

Region	Controls (n = 9) (µmol/kg Wet-Weight)	PDD Cases (n = 9) (µmol/kg Wet-Weight)	Fold-Change	*p*-Value
CB	76.7 (51.3–102.0)	45.7 (21.8–69.7)	**0.6**	**0.008**
MCX	56.9 (44.5–69.4)	45.7 (26.6–64.9)	0.8	0.3
PVC	61.1 (47.7–74.5)	54.6 (29.8–79.4)	0.9	0.5
HP	68.1 (40.2–96.0)	59.3 (26.6–91.9)	0.9	0.4
SN	71.6 (53.6–89.6)	46.0 (29.2–62.9)	**0.6**	**0.02**
MTG	47.9 (34.6–61.2)	44.2 (20.2–68.3)	0.9	0.4
MED	46.8 (38.5–55.0)	27.1 (11.5–42.6)	**0.6**	**0.008**
CG	61.3 (51.9–70.8)	46.9 (28.9–64.9)	0.8	0.1
PONS	72.2 (54.3–90.1)	48.2 (26.8–69.7)	0.7	0.0503

Mean pantothenic acid concentration with 95% confidence intervals in µmol/kg wet-weight. Case–control differences were determined using Mann–Whitney *U* test and *p* < 0.05 was considered significant. Fold-changes are cases compared to corresponding controls. See Supplementary Material B for individual data.

**Table 3:** Pantothenic Acid Concentrations in PDD Cases and Controls.

Region	Fold-Change PDD	Fold-Change AD (Xu et al., 2016)	Fold-Change HD (Patassini et al., 2015)
CB	**0.6**	**0.5**	**0.6**
MCX	0.8	**0.3**	0.6
PVC	0.9	**0.4**	0.5
HP	0.9	**0.5**	**0.5**
SN	**0.6**	-	**0.6**
MTG	0.9	**0.5**	0.6
MED	**0.6**	-	-
CG	0.8	**0.5**	0.5
PONS	0.7	-	*-*

Case–control fold-changes in pantothenic acid between three dementias. CB = cerebellum; MCX = motor cortex; PVC = primary visual cortex; HP = hippocampus; SN = substantia nigra; MTG = middle temporal gyrus; MED = medulla; CG = cingulate gyrus; PUT = putamen; GP = globus pallidus; MFG = middle frontal gyrus; ENT = entorhinal cortex. Significant intra-cohort case–control fold-changes are highlighted in bold.

## Data Availability

Raw data is available in Supplementary Material B.

## Supplementary Materials

The following are available online at https://www.mdpi.com/article/10.3390/metabo11090569/s1, Supplementary Material A: Table S1: Characteristics of individuals in the PDD cohort, Table S2: Characteristics of individuals in the PDD cohort, Table S3: Statistical Power of Pantothenic Acid Analyses, Figure S1: Pantothenic Acid Calibration Curves. Supplementary Material B: Raw Data.

## References

[1] Braak H., Del Tredici K., Rub U., de Vos R.A., Jansen Steur E.N., Braak E. (2003). Staging of brain pathology related to sporadic Parkinson’s disease. Neurobiol. Aging.

[2] Rietdijk C.D., Perez-Pardo P., Garssen J., van Wezel R.J., Kraneveld A.D. (2017). Exploring Braak’s Hypothesis of Parkinson’s Disease. Front. Neurol..

[3] Del Tredici K., Braak H. (2020). To stage, or not to stage. Curr. Opin. Neurobiol..

[4] Williams-Gray C.H., Mason S.L., Evans J.R., Foltynie T., Brayne C., Robbins T.W., Barker R.A. (2013). The CamPaIGN study of Parkinson’s disease: 10-year outlook in an incident population-based cohort. J. Neurol. Neurosurg. Psychiatry.

[5] Hely M.A., Reid W.G., Adena M.A., Halliday G.M., Morris J.G. (2008). The Sydney multicenter study of Parkinson’s disease: The inevitability of dementia at 20 years. Mov. Disord..

[6] McColgan P., Tabrizi S.J. (2018). Huntington’s disease: A clinical review. Eur. J. Neurol..

[7] Cummings J. (2020). The Role of Neuropsychiatric Symptoms in Research Diagnostic Criteria for Neurodegenerative Diseases. Am. J. Geriatr. Psychiatry.

[8] Fifel K., Videnovic A. (2020). Circadian and Sleep Dysfunctions in Neurodegenerative Disorders—An Update. Front. Neurosci..

[9] Moon Y., Sung J., An R., Hernandez M.E., Sosnoff J.J. (2016). Gait variability in people with neurological disorders: A systematic review and meta-analysis. Hum. Mov. Sci..

[10] Ding W., Ding L.J., Li F.F., Han Y., Mu L. (2015). Neurodegeneration and cognition in Parkinson’s disease: A review. Eur. Rev. Med. Pharmacol. Sci..

[11] Mestre T.A., Bachoud-Levi A.C., Marinus J., Stout J.C., Paulsen J.S., Como P., Duff K., Sampaio C., Goetz C.G., Cubo E. (2018). Rating scales for cognition in Huntington’s disease: Critique and recommendations. Mov. Disord..

[12] Gueli M.C., Taibi G. (2013). Alzheimer’s disease: Amino acid levels and brain metabolic status. Neurol. Sci..

[13] Xu J., Begley P., Church S.J., Patassini S., Hollywood K.A., Jullig M., Curtis M.A., Waldvogel H.J., Faull R.L., Unwin R.D. (2016). Graded perturbations of metabolism in multiple regions of human brain in Alzheimer’s disease: Snapshot of a pervasive metabolic disorder. Biochim. Biophys. Acta.

[14] Patassini S., Begley P., Reid S.J., Xu J., Church S.J., Curtis M., Dragunow M., Waldvogel H.J., Unwin R.D., Snell R.G. (2015). Identification of elevated urea as a severe, ubiquitous metabolic defect in the brain of patients with Huntington’s disease. Biochem. Biophys. Res. Commun..

[15] Handley R.R., Reid S.J., Brauning R., Maclean P., Mears E.R., Fourie I., Patassini S., Cooper G.J.S., Rudiger S.R., McLaughlan C.J. (2017). Brain urea increase is an early Huntington’s disease pathogenic event observed in a prodromal transgenic sheep model and HD cases. Proc. Natl. Acad. Sci. USA.

[16] Xu J., Begley P., Church S.J., Patassini S., McHarg S., Kureishy N., Hollywood K.A., Waldvogel H.J., Liu H., Zhang S. (2016). Elevation of brain glucose and polyol-pathway intermediates with accompanying brain-copper deficiency in patients with Alzheimer’s disease: Metabolic basis for dementia. Sci. Rep..

[17] Patassini S., Begley P., Xu J., Church S.J., Reid S.J., Kim E.H., Curtis M.A., Dragunow M., Waldvogel H.J., Snell R.G. (2016). Metabolite mapping reveals severe widespread perturbation of multiple metabolic processes in Huntington’s disease human brain. Biochim. Biophys. Acta.

[18] An Y., Varma V.R., Varma S., Casanova R., Dammer E., Pletnikova O., Chia C.W., Egan J.M., Ferrucci L., Troncoso J. (2018). Evidence for brain glucose dysregulation in Alzheimer’s disease. Alzheimer’s Dement..

[19] Ansoleaga B., Jove M., Schluter A., Garcia-Esparcia P., Moreno J., Pujol A., Pamplona R., Portero-Otin M., Ferrer I. (2015). Deregulation of purine metabolism in Alzheimer’s disease. Neurobiol. Aging.

[20] Martin W.R., Clark C., Ammann W., Stoessl A.J., Shtybel W., Hayden M.R. (1992). Cortical glucose metabolism in Huntington’s disease. Neurology.

[21] Xu J., Patassini S., Begley P., Church S., Waldvogel H.J., Faull R.L.M., Unwin R.D., Cooper G.J.S. (2020). Cerebral deficiency of vitamin B5 (d-pantothenic acid; pantothenate) as a potentially-reversible cause of neurodegeneration and dementia in sporadic Alzheimer’s disease. Biochem. Biophys. Res. Commun..

[22] Patassini S., Begley P., Xu J., Church S.J., Kureishy N., Reid S.J., Waldvogel H.J., Faull R.L.M., Snell R.G., Unwin R.D. (2019). Cerebral Vitamin B5 (D-Pantothenic Acid) Deficiency as a Potential Cause of Metabolic Perturbation and Neurodegeneration in Huntington’s Disease. Metabolites.

[23] Hayflick S.J. (2014). Defective pantothenate metabolism and neurodegeneration. Biochem. Soc. Trans..

[24] Scholefield M., Church S.J., Xu J., Robinson A.C., Gardiner N.J., Roncaroli F., Hooper N.M., Unwin R.D., Cooper G.J.S. (2020). Effects of Alterations of Post-Mortem Delay and Other Tissue-Collection Variables on Metabolite Levels in Human and Rat Brain. Metabolites.

[25] Lee J.H., Ahn S.Y., Lee H.A., Won K.S., Chang H.W., Oh J.S., Kim H.W. (2018). Dietary intake of pantothenic acid is associated with cerebral amyloid burden in patients with cognitive impairment. Food Nutr. Res..

[26] Baldini F., Hertel J., Sandt E., Thinnes C.C., Neuberger-Castillo L., Pavelka L., Betsou F., Kruger R., Thiele I., Consortium N.-P. (2020). Parkinson’s disease-associated alterations of the gut microbiome predict disease-relevant changes in metabolic functions. BMC Biol..

[27] Vascellari S., Palmas V., Melis M., Pisanu S., Cusano R., Uva P., Perra D., Madau V., Sarchioto M., Oppo V. (2020). Gut Microbiota and Metabolome Alterations Associated with Parkinson’s Disease. mSystems.

[28] Wang Q., Li W.X., Dai S.X., Guo Y.C., Han F.F., Zheng J.J., Li G.H., Huang J.F. (2017). Meta-Analysis of Parkinson’s Disease and Alzheimer’s Disease Revealed Commonly Impaired Pathways and Dysregulation of NRF2-Dependent Genes. J. Alzheimer’s Dis..

[29] Gibson G.E., Kingsbury A.E., Xu H., Lindsay J.G., Daniel S., Foster O.J., Lees A.J., Blass J.P. (2003). Deficits in a tricarboxylic acid cycle enzyme in brains from patients with Parkinson’s disease. Neurochem. Int..

[30] Willkommen D., Lucio M., Moritz F., Forcisi S., Kanawati B., Smirnov K.S., Schroeter M., Sigaroudi A., Schmitt-Kopplin P., Michalke B. (2018). Metabolomic investigations in cerebrospinal fluid of Parkinson’s disease. PLoS ONE.

[31] Tang B.L. (2020). Glucose, glycolysis, and neurodegenerative diseases. J. Cell Physiol..

[32] Foltynie T. (2019). Glycolysis as a therapeutic target for Parkinson’s disease. Lancet Neurol..

[33] Bohnen N.I., Koeppe R.A., Minoshima S., Giordani B., Albin R.L., Frey K.A., Kuhl D.E. (2011). Cerebral glucose metabolic features of Parkinson disease and incident dementia: Longitudinal study. J. Nucl. Med..

[34] Albrecht F., Ballarini T., Neumann J., Schroeter M.L. (2019). FDG-PET hypometabolism is more sensitive than MRI atrophy in Parkinson’s disease: A whole-brain multimodal imaging meta-analysis. Neuroimage Clin..

[35] Gonzalez-Redondo R., Garcia-Garcia D., Clavero P., Gasca-Salas C., Garcia-Eulate R., Zubieta J.L., Arbizu J., Obeso J.A., Rodriguez-Oroz M.C. (2014). Grey matter hypometabolism and atrophy in Parkinson’s disease with cognitive impairment: A two-step process. Brain.

[36] Bohnen N.I., Minoshima S., Giordani B., Frey K.A., Kuhl D.E. (1999). Motor correlates of occipital glucose hypometabolism in Parkinson’s disease without dementia. Neurology.

[37] Selnes P., Stav A.L., Johansen K.K., Bjornerud A., Coello C., Auning E., Kalheim L., Almdahl I.S., Hessen E., Zetterberg H. (2017). Impaired synaptic function is linked to cognition in Parkinson’s disease. Ann. Clin. Transl. Neurol..

[38] Ismail N., Kureishy N., Church S.J., Scholefield M., Unwin R.D., Xu J., Patassini S., Cooper G.J.S. (2020). Vitamin B5 (d-pantothenic acid) localizes in myelinated structures of the rat brain: Potential role for cerebral vitamin B5 stores in local myelin homeostasis. Biochem. Biophys. Res. Commun..

[39] Fitzgerald E., Murphy S., Martinson H.A. (2019). Alpha-Synuclein Pathology and the Role of the Microbiota in Parkinson’s Disease. Front. Neurosci..

[40] Mulak A., Bonaz B. (2015). Brain-gut-microbiota axis in Parkinson’s disease. World J. Gastroenterol..

[41] Van Den Berge N., Ferreira N., Gram H., Mikkelsen T.W., Alstrup A.K.O., Casadei N., Tsung-Pin P., Riess O., Nyengaard J.R., Tamguney G. (2019). Evidence for bidirectional and trans-synaptic parasympathetic and sympathetic propagation of alpha-synuclein in rats. Acta Neuropathol..

[42] Scheperjans F., Derkinderen P., Borghammer P. (2018). The Gut and Parkinson’s Disease: Hype or Hope?. J. Parkinson’s Dis..

[43] Braak H., de Vos R.A., Bohl J., Del Tredici K. (2006). Gastric alpha-synuclein immunoreactive inclusions in Meissner’s and Auerbach’s plexuses in cases staged for Parkinson’s disease-related brain pathology. Neurosci. Lett..

[44] Hustad E., Aasly J.O. (2020). Clinical and Imaging Markers of Prodromal Parkinson’s Disease. Front. Neurol..

[45] Gellersen H.M., Guo C.C., O’Callaghan C., Tan R.H., Sami S., Hornberger M. (2017). Cerebellar atrophy in neurodegeneration-a meta-analysis. J. Neurol. Neurosurg. Psychiatry.

[46] Wu T., Hallett M. (2013). The cerebellum in Parkinson’s disease. Brain.

[47] McKeith I.G., Dickson D.W., Lowe J., Emre M., O’Brien J.T., Feldman H., Cummings J., Duda J.E., Lippa C., Perry E.K. (2005). Diagnosis and management of dementia with Lewy bodies: Third report of the DLB Consortium. Neurology.

