# Supplementary material for: Substantively Lowered Levels of Pantothenic Acid (Vitamin B5) in Several Regions of the Human Brain in Parkinson’s Disease Dementia
Source: Metabolites. 2021 Aug 25;11(9):569. doi: 10.3390/metabo11090569 (PMC8468190; doi:10.3390/metabo11090569)
Supplement: Supplementary file 1 [file metabolites-11-00569-s001.zip › B5 Paper - Supplementary Material A (Tables) 2.0.pdf]

Supplementary Material A

Contents

Supplementary Table 1: Characteristics of individuals in the PDD cohort 2

Supplementary Table 2: Characteristics of controls in the SN cohort 4

Supplementary Table 3: Statistical Power of Pantothenic Acid Analyses........ 5

Supplementary Figure 1: Pantothenic Acid Calibration Curves 6

Supplementary Table 1: Characteristics of individuals in the PDD cohort

Code	Sex	Age at death	Clinical diagnosis	PD Neuropathology	Post-Mortem Delay (hours)	Whole-brain weight (g)	Cause of Death
C1	Male	61	No dementia present	None observed	12.5	1182	Respiratory failure; heart failure; coronary artery disease
C2	Male	71	No dementia present	None observed	23.9	1371	Acute myocardial infarction; severe coronary artery disease; pulmonary oedema; diabetes mellitus; cardiopulmonary arrest
C3	Male	74	No dementia present	None observed	25.5	1300	Aortic dissection
C4	Male	70	No dementia present	None observed	12.7	1350	Atherosclerotic and hypertensive heart disease
C5	Female	68	No dementia present	None observed	19.1	1270	Acute myocardial infarction; coronary artery disease
C6	Female	65	No dementia present	None observed	19.4	1372	Hypertensive arteriosclerotic cardiovascular disease; morbid obesity; respiratory arrest; suspected embolus
C7	Female	77	No dementia present	None observed	21.4	1135	Pending death certificate
C8	Female	79	No dementia present	None observed	17.8	1300	Acute myocardial infarction; coronary artery disease; atrial fibrillation; COPD

C9	Female	67	No dementia present	None observed	25.9	1382	Pending death certificate
PDD1	Male	61	Parkinson’s disease dementia	Limbic (transitional)	13.8	1188	Cardiopulmonary arrest; probable acute myocardial infarction; Parkinson’s disease
PDD2	Male	79	Parkinson’s disease dementia	Limbic (transitional)	16.2	1250	End stage Parkinson’s disease
PDD3	Male	71	Parkinson’s disease dementia	Brainstem predominant; Braak stage III-IV	16.2	1262	Pending death certificate
PDD4	Male	78	Parkinson’s disease dementia	Diffuse neocortical; Braak stage VI	20.4	1520	Respiratory failure; aspiration pneumonia; dysphagia; Parkinson’s disease
PDD5	Male	70	Parkinson’s disease dementia	Diffuse neocortical	4.3	1218	Aspiration pneumonia; Parkinson’s disease
PDD6	Female	69	Parkinson’s disease dementia	Braak stage IV-V	17.5	1187	Pending death certificate
PDD7	Female	81	Parkinson’s disease dementia	Diffuse neocortical	7.0	1415	End stage Parkinson’s disease
PDD8	Female	79	Parkinson’s disease dementia	Limbic (transitional)	21.9	1200	Respiratory failure; Parkinson’s disease
PDD9	Female	67	Parkinson’s disease dementia	Diffuse neocortical	14.5	Not available	Lewy body disease; Parkinson's disease

Supplementary Table 2: Characteristics of controls in the SN cohort

Code	Sex	Age at death	Clinical diagnosis	PD Neuropathology	Post-Mortem Delay (hours)	Whole-brain weight (g)	Cause of Death
C2	Male	71	No dementia present	None observed	23.9	1371	Acute myocardial infarction; severe coronary artery disease; pulmonary oedema; diabetes mellitus; cardiopulmonary arrest
C3	Male	74	No dementia present	None observed	25.5	1300	Aortic dissection
C5	Female	68	No dementia present	None observed	19.1	1270	Acute myocardial infarction; coronary artery disease
C6	Female	65	No dementia present	None observed	19.4	1372	Hypertensive arteriosclerotic cardiovascular disease; morbid obesity; respiratory arrest; suspected embolus
C7	Female	77	No dementia present	None observed	21.4	1135	Pending death certificate
C8	Female	79	No dementia present	None observed	17.8	1300	Acute myocardial infarction; coronary artery disease; atrial fibrillation; COPD
C9	Female	67	No dementia present	None observed	25.9	1382	Pending death certificate
C10	Male	68	No dementia present	None observed	10.8	1550	Pending death certificate
C11	Male	62	No dementia present	None observed	21.3	Not available	Diabetes; triple vessel disease of the heart; hyperlipidaemia

Supplementary Table 3: Statistical Power of Pantothenic Acid Analyses

Element	Statistical Power ($p < 0.05$)	Sample Size Required ($p < 0.05$)
**CB**	53.8	**6**
**MCX**	20.5	19
**PVC**	8.3	86
**HP**	7.8	99
**SN**	66.9	**4**
**MTG**	6.3	222
**MED**	73.2	**4**
**CG**	37.2	**9**
**PONS**	50.8	**6**

Bold values indicate required sample size of < 10

Supplementary Figure 1: Pantothenic Acid Calibration Curves

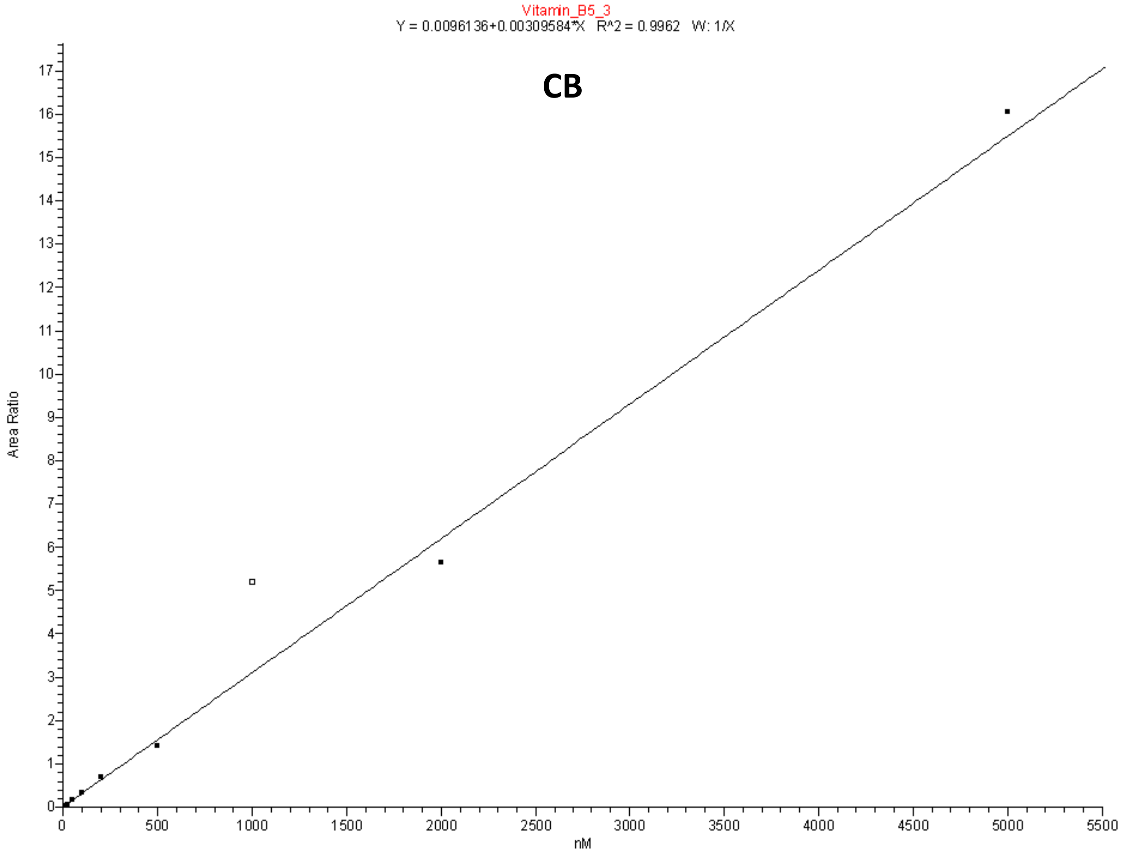

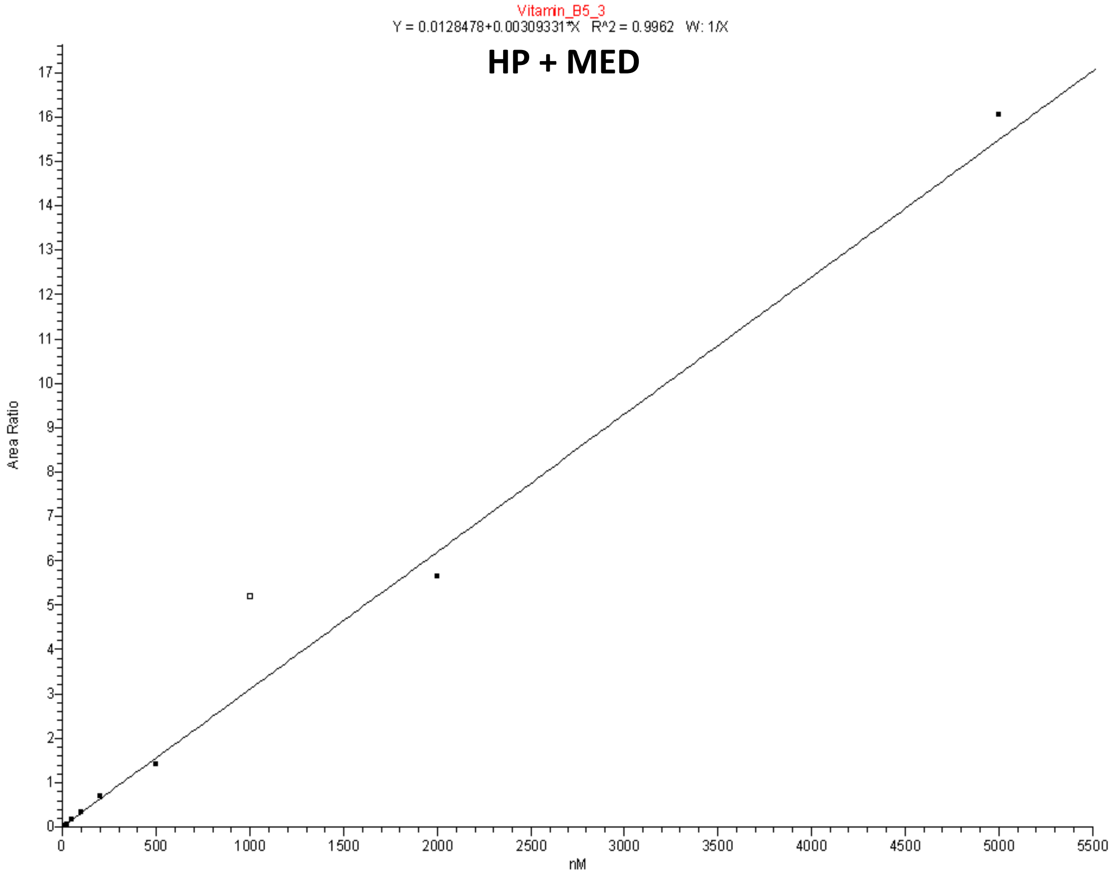

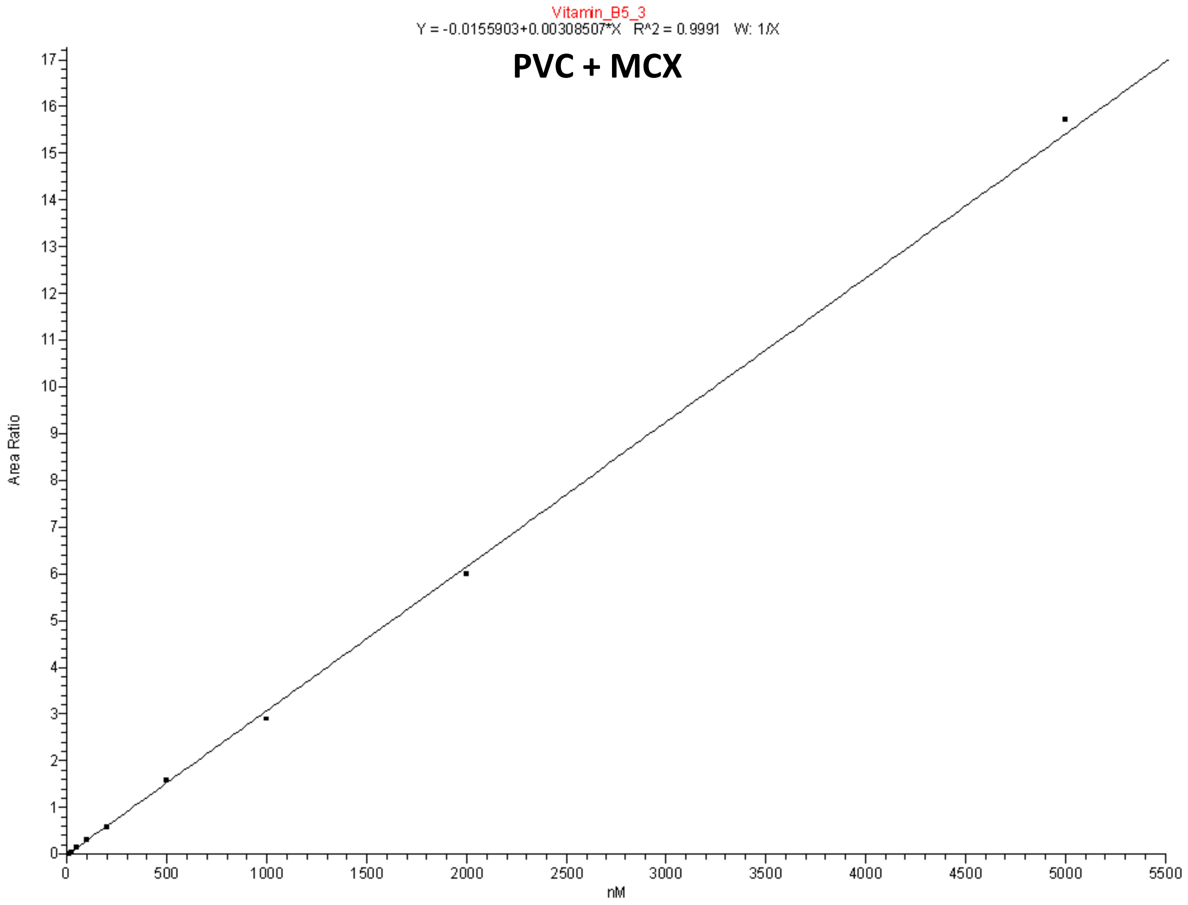
Vitamin_B5_3
Y = -0.0155903+0.00308507*X R^2 = 0.9991 W: 1/X
PVC + MCX
Area Ratio
nM

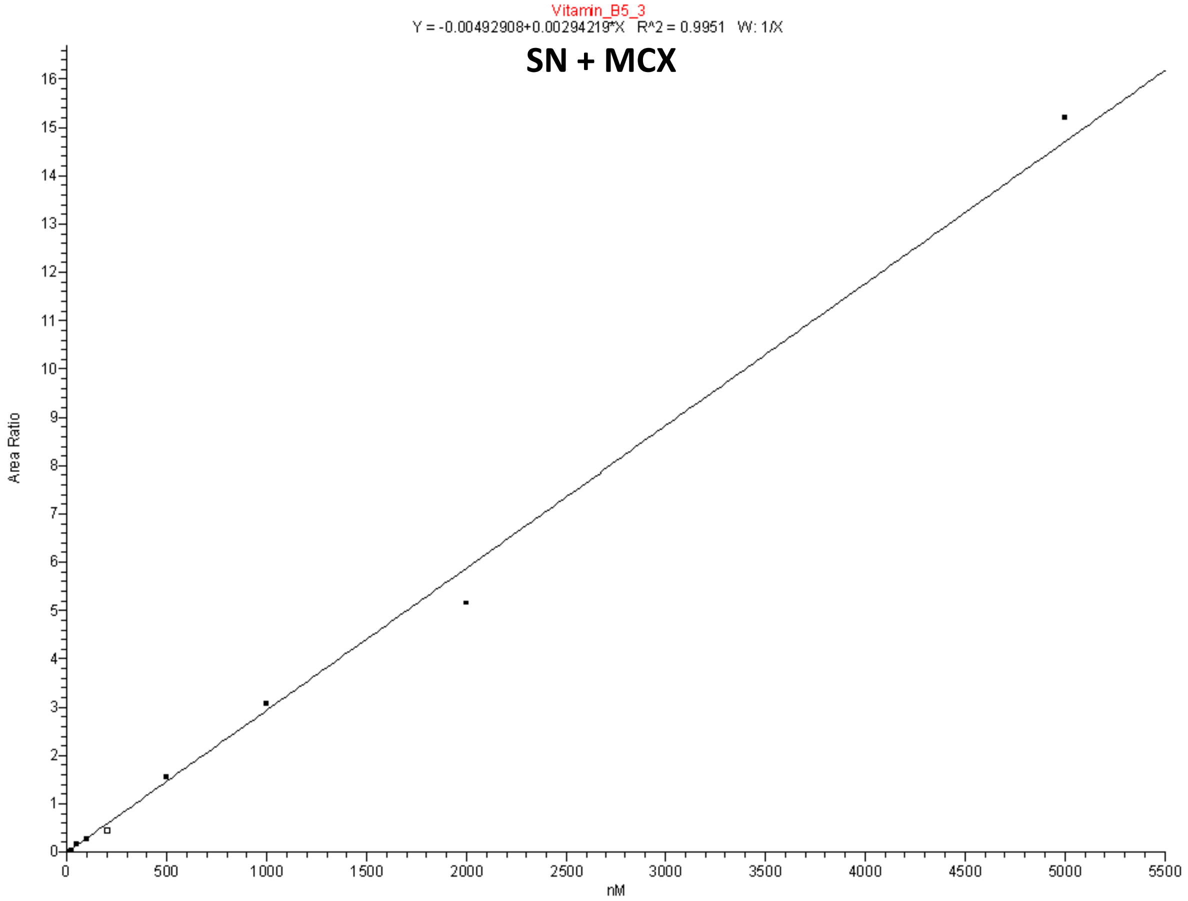
Vitamin_B5_3
Y = -0.00492908+0.00294219*X R^2 = 0.9951 W: 1/X
SN + MCX
Area Ratio
nM

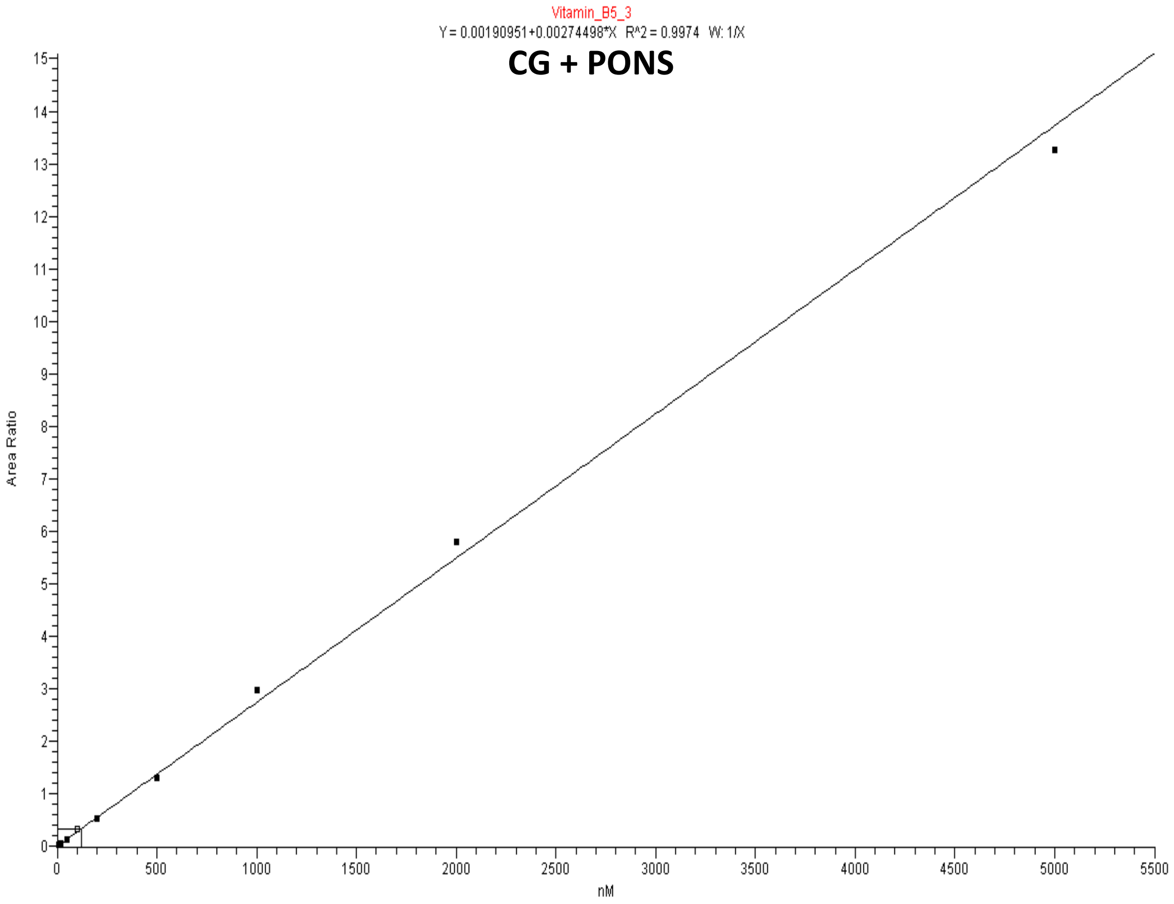

Calibration curves were generated using known concentrations of synthetic pantothenic acid standards in aqueous medium. Calibration curves were generated separately for each run.
